# Reduction of Elective Radiotherapy Treatment Volume in Definitive Treatment of Locally Advanced Head and Neck Cancer—Comparison of a Prospective Trial with a Revised Simulated Contouring Approach

**DOI:** 10.3390/jcm10204653

**Published:** 2021-10-11

**Authors:** Thomas Weissmann, Stefan Speer, Florian Putz, Sebastian Lettmaier, Philipp Schubert, Maya Shariff, Sabine Semrau, Antoniu-Oreste Gostian, Maximilian Traxdorf, Sarina K. Mueller, Markus Eckstein, Matthias Hautmann, Jens von der Grün, Marlen Haderlein, Benjamin Frey, Udo S. Gaipl, Christoph Bert, Heinrich Iro, Rainer Fietkau, Markus Hecht

**Affiliations:** 1 Department of Radiation Oncology, Universitätsklinikum Erlangen, Friedrich-Alexander-Universität Erlangen-Nürnberg, 91054 Erlangen, Germany; stefan.speer@uk-erlangen.de (S.S.); florian.putz@uk-erlangen.de (F.P.); sebastian.lettmaier@uk-erlangen.de (S.L.); philipp.schubert@uk-erlangen.de (P.S.); maya.shariff@uk-erlangen.de (M.S.); sabine.semrau@uk-erlangen.de (S.S.); marlen.haderlein@uk-erlangen.de (M.H.); benjamin.frey@uk-erlangen.de (B.F.); udo.gaipl@uk-erlangen.de (U.S.G.); christoph.bert@uk-erlangen.de (C.B.); rainer.fietkau@uk-erlangen.de (R.F.); markus.hecht@uk-erlangen.de (M.H.); 2 Comprehensive Cancer Center Erlangen-EMN (CCC ER-EMN), 91054 Erlangen, Germany; antoniu-oreste.gostian@uk-erlangen.de (A.-O.G.); maximilian.traxdorf@uk-erlangen.de (M.T.); sarina.mueller@uk-erlangen.de (S.K.M.); Markus.Eckstein@uk-erlangen.de (M.E.); heinrich.iro@uk-erlangen.de (H.I.); 3 Deutsches Zentrum Immuntherapie (DZI), Universitätsklinikum Erlangen, 91054 Erlangen, Germany; 4 Department of Otorhinolaryngologie-Head & Neck Surgery, Universitätsklinikum Erlangen, Friedrich-Alexander-Universität Erlangen-Nürnberg, 91054 Erlangen, Germany; 5 Institute of Pathology, Universitätsklinikum Erlangen, Friedrich-Alexander-Universität Erlangen-Nürnberg, 91054 Erlangen, Germany; 6 Department of Radiation Oncology, Universitätsklinikum Regensburg, Universität Regensburg, 93053 Regensburg, Germany; matthias.hautmann@ukr.de; 7 Department of Radiation and Oncology, Universitätsklinikum Frankfurt, Goethe-Universität Frankfurt, 60590 Frankfurt am Main, Germany; Jens.VonderGruen@kgu.de; 8 Translational Radiobiology, Universitätsklinikum Erlangen, Friedrich-Alexander-Universität Erlangen-Nürnberg, 91054 Erlangen, Germany

**Keywords:** de-intensification, radiotherapy, head neck cancer, definitive treatment, volume reduction, salivary glands, swallowing function

## Abstract

Definitive radiochemotherapy of locally advanced head and neck squamous cell cancer (HNSCC) achieves high locoregional tumor control rates; but is frequently associated with long-term toxicity. A future direction could be a de-escalation strategy focusing on treated volume rather than radiotherapy dose. This analysis evaluates radiotherapy dose and volume parameters of patients treated with a standard contouring approach in a clinical trial context compared with a revised volume-reduced contouring approach. In this case, 30 consecutive patients from the CheckRad-CD8 trial treated at a single study center were included in this analysis. Treatment toxicity and quality of life were assessed at the end of radiotherapy. Standard treatment plans (ST) following state of the art contouring guidelines that were used for patient treatment and volume reduced treatment plans (VRT) according to a revised simulated approach were calculated for each patient. Planning target volumes (PTV) and mean doses to 38 organs-at-risk structures were compared. At the end of radiotherapy patients reported high rates of mucositis; dysphagia and xerostomia. In addition; patient reported quality of life as assessed by the EORTC QLQ-HN35 questionnaire deteriorated. Comparing the two contouring approaches; the elective PTV_56 Gy and the high risk PTV_63 Gy (shrinking field) were significantly smaller in the VRT group. Significant reduction of mean dose to structures of the oral cavity; the larynx as well as part of the swallowing muscles and the submandibular glands was achieved in the simulated VRT-plan. Treatment de-intensification by reduction of the irradiated volume could potentially reduce treatment volume and mean doses to organs at risk. The proposed contouring approach should be studied further in the context of a clinical trial.

## 1. Introduction

Treatment of head and neck squamous cell cancer (HNSCC) is an interdisciplinary challenge. While patients with limited stage head and neck cancer are eligible for surgery, locally advanced HNSCC requires intensified radiotherapy treatment strategies. However, treatment intensification also increases the severity of acute and long-term toxicity. Despite significant improvements regarding technical issues such as the introduction of intensity modulated radiation therapy (IMRT) and volumetric modulated arc therapy (VMAT), treatment toxicity could only be reduced to a certain extent, as the radiotherapy treatment volume remained the same. The reduction of radiotherapy treatment volumes is probably an essential next step towards achieving lower toxicity. A substantially reduced toxicity was reported after radiotherapy treatment volume reduction in the postoperative setting [1]. Thus, similar approaches should be further developed in the definitive setting.

While a broad spectrum of acute and late toxicity has been reported in patients undergoing radiochemotherapy, mucositis and dysphagia seem to be the leading toxicity in the vast majority of patients [2,3,4]. While patients will in general experience an easing of their symptoms over time, a relevant number of patients will still remain dependent on their feeding tube for the rest of their lifespan due to long term dysphagia [5,6], which highlights the need for improved treatment strategies.

While chemotherapy in concomitant radiochemotherapy (RCT) improves local control by over 10% the rates of side effects are also raised by approximately 30% [7,8]. Different variations of concomitant and sequential systemic approaches including induction chemotherapy and varying intervals between chemotherapy cycles have been investigated and used in clinical practice in order to improve survival rates and minimize side effects. Attempts to deescalate systemic therapies to less aggressive regimes in selected subgroups have often proved to be inferior in terms of progression-free as well as overall survival. Prospective studies carried out by Gillison as well as Mehanna et al. failed at attempting treatment de-escalation through replacing cisplatinum by cetuximab in HPV-positive HNSCC [9,10]. The introduction of immunotherapy has sparked expectations of an oncologic break-through with dramatic improvements in terms of side effects and survival rates in the recurrent/metastatic stage, while convincing results in the curative setting and in combination with radiotherapy in particular are still pending [11].

Technical achievements in the field of diagnostics regarding the integration of MRI and FDG-PET/CT have improved initial staging, treatment planning as well as post-treatment surveillance. Especially the detection of involved lymph nodes is improved by FDG-PET/CT [12], which reduces the risk of non-detected lymph node metastases. This method not only holds the potential to measure metabolism, but also contributes to a better identification of the gross tumor volume in the planning process and holds a true potential to optimize volume and dose prescription in radiotherapy.

Among the different options available, the reduction of the electively irradiated volume is potentially the most appropriate approach to optimize treatment planning. A vast amount of data has been published over time illustrating the varying incidence of involvement of different lymph node levels. While Levels II and III show the highest frequency of lymph node involvement, Level I, Level IV and Level V seem to be involved significantly less often depending on primary tumor localization [13,14,15]. This very fact may open the possibility to reduce treatment volume. Hence in the current study a new approach for the delineation of the elective treatment volume in definitive RCT was developed aiming towards reduction of the electively treated volume through the omission of less frequently involved lymph node levels, i.e., especially levels I and IV in an attempt to lower short- and long-term toxicity. The elective inclusion only of the most frequently affected levels for each primary localization and in case of actual involvement of a certain lymph node level inclusion of the adjacent caudal level would intuitively appear to be a safe approach towards reducing the cranial and caudal field extension. Following this approach and applying the highest standards in pre-diagnostics, this concept could potentially even be applied safely in patients with caudally located primary tumors such as hypopharyngeal carcinoma for example. This new contouring approach was dosimetrically simulated and compared to a cohort of patients treated with standard contouring in the prospective CheckRad-CD8 trial. To achieve this, the actually delivered treatment plans were compared to the revised treatment plans regarding the dose to critical organs-at-risk.

## 2. Materials and Methods

### 2.1. Design of the Study

The aim of the current study is to evaluate to which extent radiation dose to the organs at risk can be reduced by a newly developed volume-reduced contouring approach when compared to a standard contouring approach. The first 30 consecutive patients treated in the CheckRad-CD8 study at a single center were screened for this analysis. The first 30 patients by order of enrollment were chosen so as to exclude a possible selection bias. Five patients were not eligible for the present analysis either due to their undergoing surgery after induction chemo-immunotherapy or their refusal to participate in additional research projects beyond the original clinical trial and therefore had to be replaced by the subsequent five patients. In total 30 patients out of the first 35 patients of the CheckRad-CD8 trial treated at a single center were included in the current analysis. All patients received a single cycle of induction treatment consisting of cisplatinum/carboplatinum in combination with docetaxel and dual immune checkpoint blockade. Dual immune checkpoint blockade consisted of the PD-L1 inhibitor durvalumab and the CTLA-4 inhibitor tremelimumab. Data on the efficacy and safety of this induction therapy has been reported before [16]. Depending on pathologic treatment response patients either continued with radioimmunotherapy in the trial or went on to receive standard radiochemotherapy outside the trial. In all patients the radiotherapy treatment volumes were contoured according to the CheckRad-CD8 trial, whose guidelines of contouring are closely similar to the current administration trial Keynote-412 following state of the art treatment recommendations [17,18,19,20]. The delivered radiotherapy treatment plan was analyzed as the standard approach in the current plan comparison study. Based on current literature a new volume-reduced radiotherapy contouring approach was developed and simulated for the selected patient cohort. The main difference is to abandon the definition of the elective nodal volume by N-stage in favor of an approach focusing on involved and adjacent lymph node levels (see Table 1). This new strategy reduces the elective nodal volume especially in patients with low a low degree of lymph node involvement. The literature leading to this approach is given in the discussion.

### 2.2. Trial Oversight

The CheckRad-CD8 trial was registered with ClinicalTrials.gov (identifier: NCT03426657). The leading institutional review board at the Friedrich-Alexander-Universität Erlangen-Nürnberg (number: 131_18 Az) approved the trial. All patients gave written informed consent to participate in the trial and for the use of their clinical and imaging data for further research projects. The trial was conducted as an investigator sponsored trial (IST).

### 2.3. Organs at Risk

For all patients included in the present study organs at risk were contoured in accordance with standards set out in high impact publications such as those by Christianen et al. [21] and Brouwer et al. [22]. The contoured structures included neurologic structures such as the brain, auditory nerves, optic chiasm, cochleae, the eyes, the lens, the optic nerves, the spinal cord, the brainstem and brachial plexus. In addition to the oral cavity itself, several associated structures such as the buccal mucosa, the mandible, the temporomandibular joints, the lips and the soft palate were delineated. Furthermore swallowing-related structures including the base of tongue (BOT) the superior, middle and inferior pharyngeal constrictor muscles (PCM), as well as the crycopharyngeal, the esophagus inlet muscle (EIM) and the cervical esophagus were depicted. The present study included the delineation of the lacrimal, the parotid, the sublingual and the submandibular glands. Furthermore, vascular structures including the carotids, the glomus caroticum as well as laryngeal structures including the supraglottic, the glottic larynx and the arytenoids were drawn. Endocrinologic structures such as the pituitary gland and the thyroid were also considered as well as muscular structures related to trismus including the M. pterygoideus medialis/lateralis M. temporalis and M. masseter. For paired structures both sides were contoured separately.

### 2.4. Contouring Guidelines

**Figure 1:**
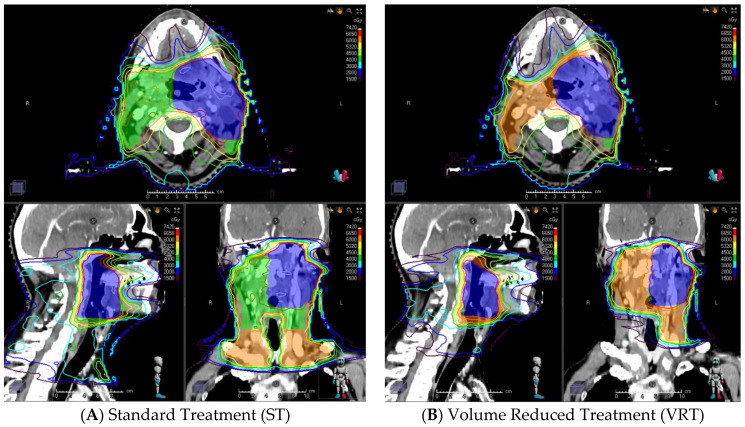
A Patient with an oropharyngeal carcinoma cT4 cN2b cM0 and lymph node involvement of level 2 on the left side. While PTV_70 (high dose volume; blue) shows identical extension, PTV_63 (intermediate dose volume; green) and PTV_56 (elective volume, orange) show a significant reduction in treated volume. The present example clearly depicts the involvement of levels 2–4 bilaterally in the standard treatment arm (**A**) while the plan from the simulated reduced volume arm (**B**) is seen to end caudally at level 2 on the right side and limited to levels 2 and 3 on the left side. The reduced-volume plan (**B**) achieves a considerable dose reduction to caudal anatomical structures potentially resulting in reduced short- and long-term toxicity.

For all patients a new volume reduced treatment plan (VRT) was calculated and compared to the delivered standard treatment plan (ST). Patients did not truly undergo radiation treatment according to the VRT plan but simulated data was used to predict dose reduction to OAR. Both contouring approaches are given in Table 1. Lymph node levels were defined according to Gregoire [23]. Contouring of gross tumor volume (GTV), clinical target volume (CTV) and subsequent planning treatment volume (PTV) was carried out using syngo.via (Siemens Healthineers, München, Deutschland). Planning target volume (PTV) was calculated from clinic target volume (CTV) adding a safety margin of 5 mm. An example is illustrated in Figure 1.

### 2.5. Radiotherapy Treatment Planning and Dose Calculation

Dosing of radiotherapy was 70.0/63.0/56.0 Gy delivered in 35 fractions to the gross tumor volume (PTV_70), high risk nodal volume (PTV_63) and elective nodal volume (PTV_56). All structures were uploaded to the Raystation planning system (Raysearch, Stockholm, Sweden, Version 9B) for dose calculation. For statistical analysis a Phyton script was written to transmit selected dose constraints to MS Excel (Microsoft, Albuquerque, NM, USA). In patients without lymph node involvement PTV_63 was generated as a copy of the PTV_70 (Boost). Dose parameters that were extracted from both planning approaches were Dmax and Dmean of all the organs at risk contoured. Calculations were carried out separately for paired structures. Both treatment plans were calculated using a simultaneously integrated boost (SIB) approach. All planning CTs were acquired in 3 mm slices using contrast agent if feasible.

### 2.6. Evaluation of Toxicity and Patient Reported Outcome

Toxicity and patient reported outcome (PRO) was prospectively documented for all patients. For patients continuing treatment within the CheckRad-CD8 trial toxicity and PRO was also prospectively documented at the end of radiotherapy. As some patients did not continue study treatment and received RCT, their toxicity at the end of radiotherapy was not analyzed. Data acquisition on the last day of radiotherapy was chosen to detect the highest level of toxicity. Toxicity was documented according to CTCAE v4.03 and PRO was obtained using the EAT-10, the EORTC QLQ-30 and the EORTC HN-35 questionnaires [16,24].

### 2.7. Statistical Analysis

The t-test for dependent samples was used to calculate differences for each organ at risk as well as for the different treatment volumes between the two contouring strategies. Calculations were carried out using IBM SPSS software for MS Windows (SPSS Inc. Chicago, IL, USA, version 21). *p* values ≤ 0.05 were considered statistically significant. To adjust for alpha error the Benjamini and Yekutieli procedure for the decrease of the false discovery rate was applied [25,26]. Graphical illustration of the distribution of values for each organ at risk as well as treatment volume for both treatment plans was carried out through boxplots using graph pad prism (GraphPad Software, SanDiego, CA, USA, Version 8).

## 3. Results

### 3.1. Patients Characteristics

Patient characteristics of the 30 included patients are given in Table 2. Out of the patients who received induction chemo-immunotherapy in the CheckRad-CD8 study, 21 patients (70%) completed radioimmunotherapy. An additional seven patients did not start radioimmunotherapy due to raised serum transaminases/hepatitis (*n* = 3), patients’ choice for alternative treatment (*n* = 3) or no significant intratumoral CD8+ immune cell increase (*n* = 1). One patient discontinued concomitant immunotherapy during radiotherapy due to increased transaminases/hepatitis and one patient discontinued radiotherapy of his own accord without toxicity. Safety and feasibility of induction treatment of the CheckRad-CD8 trial was reported before [16]. All 30 patients were included in the radiotherapy treatment plan comparison, whereas toxicity data were only available for the 21 patients who completed radioimmunotherapy as described in Table 2.

### 3.2. Side Effects on Last Day of RT

21 out of the 30 patients (70%) included had data on toxicity and PRO available for evaluation concerning their clinical status on the last day of RT. The main radiotherapy related grade II-III toxicity was mucositis at 52% (11/21), xerostomia at 43% (9/21) and dysphagia at 95% (20/21). The EAT 10 questionnaire showed a mean score of 23.4 (SD 11.8) at last RT and a change of +17.4 (SD 12.4) compared to baseline which equates to a severe worsening. As the QLQ-30 does not focus clearly on radiotherapy-related side effects, it was not included in the current analysis. The QLQ-HN35 questionnaire, which more closely relates to certain anatomic structures additionally highlighted impaired speech, sensory impairment as well as difficulties swallowing, compromised social eating and social contacts in general as a major burden. According to QLQ-HN35 patients also suffered to a considerable extent from increasing problems with mouth opening. They also reported deterioration regarding dry mouth, sticky saliva, coughing and a strong dependence on pain killers at last RT. More than half of the patients showed a dependence on tube feeding and the majority of patients had experienced weight loss over the course of their therapy. All side effects are described in Table 3.

### 3.3. Reduction in Treatment Volume

**Figure 2:**
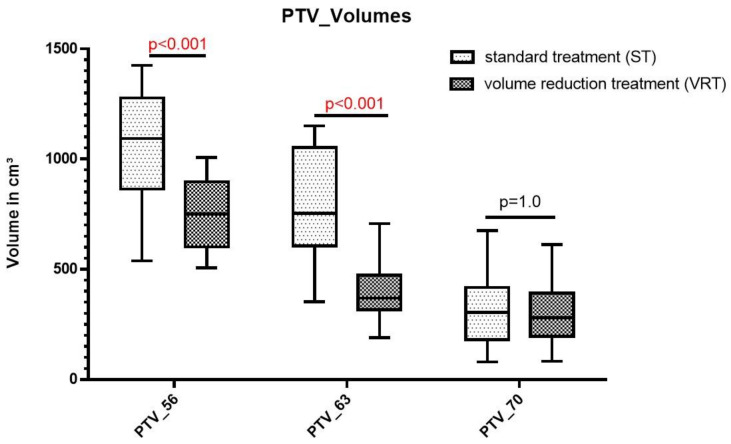
Box-Whisker-Plot comparing the different PTV_Volumes. While significant dose reduction is achieved for PTV_56 and PTV_63 applying the simulated volume reduction treatment, no significant differences were observed for PTV_70 between the standard treatment and the volume reduced treatment.

Dose-Volume calculations show a significant reduction in the elective radiotherapy treatment volume PTV_56 (median 1091.9 cm^3^ vs. 750.3 cm^3^; *p* < 0.001) and the high-risk elective PTV_63 (shrinking field; median 754.3 cm^3^ vs. 368.77 cm^3^; *p* < 0.001) for the volume-reduced treatment plan (VRT) compared to the standard treatment plan (ST). Treatment volume for PTV_70 covering the gross tumor volume with safety margin did not differ significantly between treatment groups (median 304.7 cm^3^ vs. 279.9 cm^3^; *p* = 1.000). Figure 2 shows an improved result in volume reduction using the modified contouring approach. 

### 3.4. Dose Comparison in Organs at Risk

While dose measurements for 38 different structures were carried out, only changes of the mean dosage (Dmean) for both treatments are presented in the following section to simplify presentation and facilitate the understanding of the dosage to the organs at risk. A more extensive presentation of doses (Dmean) to the investigated structures is given in the supplement section.

**Figure 3:**
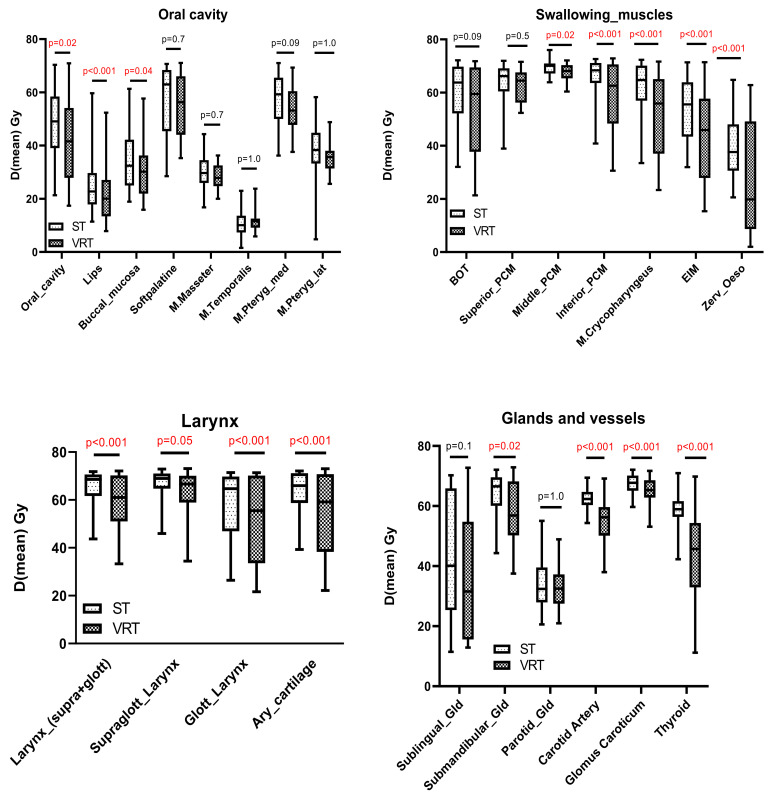
Box-Whisker-Plot comparing the different mean dosages for four different groups of organs at risk comparing standard treatment (ST) and simulated volume reduced treatment (VRT). Graph show median + IQR and statistical analysis was carried out via students t-test for dependent samples.

Dose comparisons in organs at risk are presented in the groups oral cavity, swallowing muscles, larynx and salivary/endocrine glands as presented in Figure 3.

In addition to the mean dosage to the oral cavity itself (Dmean median 49.1 Gy vs. 41.6 Gy; *p* = 0.02) significantly lower dosages were found for the lips (Dmean median 22.7 Gy vs. 20.1 Gy; *p* < 0.001) and the buccal mucosa (Dmean median 32.5 Gy vs. 30.2 Gy; *p* = 0.037) but not M. pterygoideus medialis/lateralis (Dmean median 59.6 Gy vs. 53.2 Gy *p* = 0.099/Dmean median 38.3 Gy vs. 35.7 Gy; *p* = 1.000) or the soft palate (Dmean median 62.9 Gy vs. 56.3 Gy; *p* = 0.692). While significantly lower Dmean doses were calculated for the lower swallowing muscles including the middle PCM (Dmean median 70.1 Gy vs. 68.1 Gy; *p* = 0.020) inferior PCM (Dmean median 68.3 Gy vs. 62.6 Gy; *p* < 0.001), the crycopharyngeus muscle (Dmean median 64.7 Gy vs. 55.9 Gy; *p* < 0.001), the EIM (Dmean median 55.6 Gy vs. 45.9 Gy; *p* < 0.001) and the cervical esophagus (Dmean median 37.6 Gy vs. 19.8 Gy; *p* < 0.001), no significant difference was measured for the upper structures including the BOT (Dmean median 63.8.6 Gy vs. 59.5 Gy; *p* = 0.086) and the superior PCM (Dmean median 66.2 Gy vs. 64.5 Gy; *p* < 0.541). For laryngeal structures a dose reduction for the supraglottic and glottic larynx (Dmean median 68.6 Gy vs. 61.1 Gy; *p* < 0.001) and the arytenoid cartilages (Dmean median 65.9 Gy vs. 59.1 Gy; *p* = 0.046) was detected. Furthermore, significant dose reduction was achieved in the carotid artery (Dmean median 62.4 Gy vs. 56.2 Gy; *p* < 0.001), the glomus caroticum (Dmean median 62.4 Gy vs. 56.2 Gy; *p* < 0.001) and the thyroid gland (Dmean median 58.9 Gy vs. 45.7 Gy; *p* < 0.001). Regarding the salivary glands, only the submandibular glands (Dmean median 66.6 Gy vs. 56.8 Gy; *p* < 0.001) showed a reduction in mean dose. The dose distribution for each structure and group is presented in Table 2. The complete list of all median Dmeans and interval from quartile 25%-quartile 75% is given in the Supplementary Materials Table S1.

## 4. Discussion

Reduction of treatment intensity in radiotherapy treatment of HNSCC has been subject to extensive research. Different approaches have been followed including modification and dose reduction of concomitant systemic therapies, induction treatment, changes in radiation dosages as well as radiation volume reduction for patients undergoing radiotherapy in the definitive or the adjuvant setting [1,27,28]. In the definitive setting several approaches have been followed to optimize treatment outcome. Hybrid approaches such as MRI/PET guided GTV delineation in HNSCC have been reported to show great potential to improve tumor delineation [29]. Furthermore FDG-PET/CT and 3T-MRT has dramatically improved the detection of involved levels of the head and neck and the detection of occult metastases [12,30,31,32].

In general, two approaches seem to be reasonable for dose de-escalation. These are de-escalation applying lower dosages on the one hand and reduction of radiation volumes on the other. While successful dose reductions have been shown for selected patient populations with favorable-risk HPV-positive oropharyngeal HNSCC, studies such as Johansen et al. as well as Tandon et al. have shown the majority of recurrences occurring in high dose areas questioning that dose reduction is a reasonable option in unselected general population [33,34,35].

So far, several trials have been carried out aiming towards treatment de-escalation. Villaflor et al. as well as Seiwert et al. have successfully proven the feasibility of dose and volume reduction following induction chemotherapy in the definitive treatment of HNSCC for HPV-positive preselected and unselected populations and achieved encouraging treatment results [36,37]. In contrast, radiotherapy treatment planning according to the KEYNOTE-412 study protocol which currently is regarded as the international treatment standard as well as general contouring guidelines seem to be very conservative even when the actual tumor bulk appears to be limited. KEYNOTE-412 treatment for example recommends bilateral treatment of the neck even when nodal involvement is limited to one side. Furthermore, irradiation of all consecutive levels of one side is recommended even for cases where only one apical level such as for example level II is involved. In this context it is worth mentioning Villaflor et al., who limited the radiotherapy treatment volume to the gross tumor volume with safety margin in good responders to induction therapy and who saw the majority of recurrent tumors (12/13) within the radiation field and (11/12) even in the high dose field questioning the need for larger treatment volumes. According to KEYNOTE-412 contouring guidelines as well as general contouring guidelines [17,18,19,20] the elective treatment volume is determined rather by N-stage status and less by the primary tumor localization potentially leading to large treatment fields. This generally accepted approach seems old fashioned as lymph node affection can be expected to originate from tumor spreading from its initial localization to adjacent lymph node levels and to continue its expansion from there. This is why the presented modified contouring approach provides information on certain levels that have to be included that are specific to each tumor localization as suggested by Biau and Gregoire, opening the possibility for volume reduction in low risk areas. Our approach is based on several studies investigating the distribution of pathological cervical lymph nodes in carcinomas of the oral cavity, the oropharynx and the hypopharynx. A study by Deo et al., for example shows a level III involvement twice as high and a Level II involvement eight times as high as compared to Level IV (16.6% vs. 8.6%; 52.4% vs. 8.6%) involvement in a study with 945 patients with cancer of the oral cavity. Level III itself is affected three times less frequently when com-pared to level II (16.6% vs. 52.4%) and four times less frequently when compared to level I (16.6 vs. 60.9%). The probability of isolated involvement of Levels III or IV without affection of the more cranial levels therefore can be expected to be even lower justifying a reduction of treatment volume in the lower segments in selected patients [13]. A study by Sanguinetti et al. thoroughly investigates the distribution of level involvement in oropharyngeal cancer. The study describes an estimated risk of 5–7% of Level IV involvement rising to around 11% if level III is involved on computed tomography imaging. In addition, the study further describes a probability distribution of lymph node involvement with reduced probability of involvement for more caudal locations. While Level I shows low rates of involvement of around 4% and might therefore safely be omitted from the treatment volume the high rates of involvement of up to 91% for level II require its unconditional inclusion in the treatment volume in all cases. The high incidence of involvement of Level II would automatically lead to the inclusion of Level III in the vast majority of cases. The classical approach including Levels II, III and IV even in nodal negative patients would appear to us as overtreatment. These results in our opinion highlight the potential to re-duce treatment volume both at the cranial (Level I) and caudal borders (Level IV) in oropharyngeal carcinoma as well [14]. While hypopharyngeal carcinomas show a very low rate of affection of Level IV in nodal negative imaging, involvement rises significantly up to 40% in patients with nodal positive imaging [15]. To account for this elevated risk our contouring plan is expanded by including the adjacent uninvolved lymph node level below consequently including all levels on one side of the neck if necessary. On the other hand, level I is also very rarely affected especially in negative imaging showing an affection in only 2% of the cases supporting the concept of reducing the target volume in this area.

Another topic that has to be critically discussed is the occurrence of skip lesions, meaning involvement of lower nodal stations without the involvement of preceding nodal levels ranging from 0–5% [15,38,39]. Intensified utilization of state of the art pretherapeutic di-agnostics especially FDG-PET-CT and MRI holds a true potential for addressing just these cases and minimizing the residual risk of missing pathological lymph nodes in treatment planning limited to CT. The present contouring approach is designed to combine treatment safety with the potential to reduce side effects by reducing the elective radiotherapy treatment volume as effectively as possible.

Regarding treatment results our modified contouring guidelines have met several important goals. While the reduction in PTV_56 (56 Gy) and PTV_63 (63 Gy) was expected, we quantified the simulated dose reduction in a variety of organs at risk potentially easing the short- and long-term side effects for patients.

A significant dose reduction for a broad variety of organs at risk was detected. Regarding trismus, it is unfortunate in view of its strong contribution to the toxicity seen following radiation therapy as described in the literature and supported by the findings from our own cohort, that the mean dose reduction did not translate to a significant dose reduction to the pterygoid muscles, the M. temporalis and M. masseter which have been associated with trismus in patients undergoing radiotherapy [40]. Another important side effect in our patient cohort, which is also frequently reported in the literature, is the high rate of xerostomia after radiotherapy, contributing to dysphagia with subsequent malnutrition or even speech deficits [41]. Therefore, improvements regarding this topic should be viewed as a priority especially considering our own prospective data showing the highest values of worsening for xerostomia and sticky saliva. It is a fact that the mucosa of the oral cavity itself contains around 1000 smaller glands participating in the total gross production of around 0.5 up to 1.5 L of salivary fluids per day. It is therefore an important finding that our contouring strategy yields a significant reduction of the mean dose to the oral cavity as well as the submandibular glands which according to NTCP-modelling studies potentially reduces the risk of severe xerostomia [42]. While data is available suggesting that dosages above mean dosages of 39 Gy to submandibular glands are associated with significantly worse results for xerostomia, we have to point out that dosages lower than 39 Gy were achieved in our patient cohort. We preferred to report mean dosages to paired organs combined because mean doses to the salivary glands can be significantly different between the two sides especially in lateralized tumors [43] which together with low patient numbers and the heterogeneous localization of the primary could potentially lead to significant bias in case of separate reporting of mean doses. Even though our results, unfortunately fail to show a possible benefit in the parotids, the sublingual gland which is mainly responsible for the production of the resting saliva-at least shows a tendency towards lower dosages despite not being significant. In addition, we expect that the reduced dose to the oral cavity as well as the dose to the buccal mucosa and the lips will reduce the rate of severe mucositis, which severely affected over 50% of patients at the time of their last RT session in our analysis.

Swallowing dysfunction is a main consequence of definitive radio(chemo)therapy of HNSCC also reported in over 90% of the patients in our cohort and clearly represented by the striking increase of the EAT score [44]. While different approaches have been dis-cussed, side effects due to the high doses required in the definitive treatment seem to be inevitable [45,46]. Our results do not show a possibility for dose reduction to the cranial swallowing structures including the BOT and the superior constrictor pharynges, as a consequence of the superior goal of maintaining tumor control as high as possible.

Nevertheless, we found reduced doses to the lower swallowing structures including the middle and inferior PCM, the crycopharyngeus muscle, the EIM and the cervical esophagus. This according to NTCP-models offers a chance to reduce swallowing side effects and potentially even the long-term dependency on a feeding tube with the associated high rates of weight loss during therapy, which was also detected in our trial [47,48]. Lower dosages to the esophagus have the potential to reduce esophageal stenosis that is frequently reported in head and neck irradiation [49,50]. While laryngeal structures are of great importance for swallowing they also play a significant role in voice production. The reduction of mean dose to the supraglottic as well as glottic larynx has the potential not only to improve voice quality as suggested by NTCP models [51] but also to improve swallowing. Impairment of swallowing frequently leads to aspiration and choking not rarely resulting in pulmonary infections [52]. The dose reduction to the cartilageanous structures including the arytenoid cartilages and the thyroid cartilages in particular has the potential to reduce necrosis, fracture or symptoms of hoarseness [53,54,55].

Long term side effects to vascular structures are generally little noticed. Nevertheless, it is well known that RT in the treatment of HNSCC can cause carotid stenosis doubling the risk for neurological events including transient ischemic attack (TIA) or ischemic stroke [56,57,58]. At the moment reducing dose to the vascular system is not the objective of treatment planning optimization in patients with HNSCC due to the close proximity of the vascular system and the lymphatic drainage of the neck. While different rates of stenosis have been reported for the different branches of the carotids we chose the glomus caroticum as a representative structure [58]. The present study was able to show a significant dose reduction to the glomus caroticum as well as the full length of the carotid artery raising the possibility of a true benefit for patients surviving long-term.

Many patients develop hypothyroidism following radiotherapy due to several mechanism including vascular effects, fibrosis and damage to the follicular epithelial cells potentially leading to severe side effects such as cardiovascular, neurosensory and psychological conditions [59,60]. Our results clearly show that the new contouring guidelines could significantly reduce dosage to the thyroid, reducing risk of organ failure in the long term with the associated need for lifelong hormone replacement.

In summary we were capable of showing that applying our newly developed contouring approach has the potential to reduce radiation dose to several important organs at risk potentially benefitting patients regarding short- and long-term side effects. Especially for the more caudally located structures dose reductions have been achieved which can truly be expected to benefit the patient. The present contouring strategy might even allow further treatment de-escalation such as further steps of dose de-escalation, but is not tailored to favorable subgoups of patients such as HPV-positive patients but rather laid out to meet the need of the majority of HNSCC patients.

Nevertheless, several limitations have to be discussed. First of all, the present contouring approach has not been prospectively studied. There is at least to some extent a higher risk of tumor recurrence that cannot be discounted. However, the majority of recurrences in volume reduction studies have been reported within the radiated field and even the high dose fields questioning the need for very extensive elective radiation fields especially when making extensive use of state-of-the-art pretreatment diagnostics. Another drawback in the present volume reduction trial is that despite the achievement of significant dose reduction in several structures one has to remember that the respective organs at risk still receive significant dosages causing short- and long-term side effects. However, we expect the calculated dose reductions to result in a real clinical benefit for the patients, especially considering potential compensation mechanisms between different OAR.

Furthermore, the high-risk elective volume PTV_63 (63 Gy) through the lack of a generally accepted definition is still a matter of debate. No consensus on how to define this volume exists to date. Definitions of this volume range from the PTV_56 without the supraclavicular area, through an involved field concept treating only the involved lymph node levels, to an involved node concept treating only the macroscopic tumor volume with larger safety margins.

## 5. Conclusions

In conclusion, the presented contouring approach holds the potential to reduce treatment side effects without risking high rates of tumor recurrence. The inclusion of FDG-PET/CT and/or MRI or even radiomic biomarkers should be considered to compensate for potential higher recurrence risk due to lower treatment volumes. Patients may also be preselected for such a de-escalation strategy, which might be performed via induction chemotherapy [36,37,61,62,63]. The present study could be a first step towards a more individualized treatment strategy modifying and optimizing radiotherapy treatment volume, that will need to be investigated within the framework of a prospective trial.

## Figures and Tables

**Table 1:** Comparison of contouring guidelines for standard treatment (ST) and volume reduced treatment (VRT). GTV, gross tumor volume; CTV, clinical target volume; RP, retropharyngeal; RS, retrostyloid.

	Standard Treatment (ST)			Volume Reduced Treatment (VRT)	
CTV_70	GTV + 1 cm			GTV + 1 cm	
**CTV_63**	minimum including involved lymph node levels; in any case: excluding medial supraclavicular lymph nodes			involved lymph node levels	
**CTV_56**	GTV + 1.0 cm			GTV + 1.0 cm	
	**Nodal stage (TNM 7)**	**Ipsilateral**	**contralateral**	**Primary tumor**	**Include bilateral level**
	N0-1	II, III, IV	II, III, IV	Oral cavity	Ib, II
	N2a-N2b	Ib, II, III, IV, V + RP	II, III, IV	Oropharynx	II
	N2c	Ib, II, III, IV, V + RP	according N contralateral	Hypopharynx	II, III
	N3	I, II, III, IV, V + RP	according N contralateral	Larynx	II, III
	Level VI: any primary site with trans- or subglottic extension or extension to pyriform sinus or esophagus			**Always include the level caudal to an involved level** (Level IVa and IVb count as separate levels). Include Level VIa in case of transglottic invasion. Include Level VIb for trans- or subglottic extension or extension to pyriform sinus or esophagus Include bilateral level V if level II, III or IV included (cranio-caudal extension of level V should not exceed beyond the involved level)	
**Peculiarities:** Include Ia for involved anterior tongue and anterior base of mouth Include Ib if oral cavity is involved. Include bilateral medial and lateral retropharyngeal (RP; VIIa) and retrostyloid (RS; VIIb) if level II is involved. Include RP (VIIa): for all primary tumor crossing the midline and in all cases with primary tumor extension to the posterior pharyngeal wall irrespective of N stage and all oropharyngeal tumors. Medial Supra-clavicular fossa nodes: systematic irradiatation of the supra-clavicular fossa lymph nodes in case of Level IV nodal infiltration is recommended. Include 2 cm of the sternocleidomastoid muscle in case of tumor infiltration					
The TNM 7th edition of the AJCC Cancer Staging Manual was applied for radiotherapy treatment planning.					

**Table 2:** Patients characteristics.

Patient Characteristics	No. (*N* = 30)	%
Age (median, SD)	60.5 ± 9.1	
Sex		
Male	22	73
Female	8	27
ECOG-Status		
0	23	77
1	7	23
Primary tumor site		
Oral cavity	1	3
Oropharynx	15	50
Hypopharynx	8	27
Larynx	6	20
T-category		
T1	2	7
T2	5	17
T3	4	13
T4	19	63
N-category		
N0	5	17
N1	8	27
N2	11	37
N3	6	20
Tobacco smoking		
Current smoker	15	50
Former smoker	10	33
Never smoker	5	17
Pack years of current/former smokers (mean, SD)	40.0 ± 19.8	
Level involvement		
Level I	6/0	20/0
Level 2 uni/bilateral	16/7	53/23
Level 3 uni/bilateral	16/0	53/0
Level 4 uni/bilateral	3/1	10/3

**Table 3:** Side effects at last RT.

Side Effects at Last RT		
***CTCAE***	Stage	
Mucositis	Grade 2 + 3	52% (11/21)
Dysphagia	Grade 2 + 3	95% (20/21)
Xerostomia	Grade 2 + 3	43% (9/21)
Radiodermatitis	Grade 2 + 3	76% (16/21)
***EAT 10***	At last RT: Mean 23.4 SD 11.8 Comparison to baseline: +17.4 SD 12.4	
**QLQ-HN35**	Comparison to baseline: Mean/SD	
Pain	+31.1/25.6	
Swallowing	+33.6/28.4	
Sensory problems	+48.2/26.6	
Speech problems	+29.8/22.8	
Trouble with social eating	+54.4/31.7	
Trouble with social contact	+18.1/23.2	
Less sexuality	+30.1/39.7	
Teeth	+5.0/27.1	
Opening mouth	+37.0/41.0	
Dry mouth	+47.6/32.6	
Sticky saliva	+64.9/38.2	
Coughing	+38.1/25.7	
Felt ill	+31.4/32.2	
Pain killers	+37.5/61.9	
Nutritional supplements	+53.3/51.6	
Feeding tube	+75.0/44.7	
Weight loss	+33.3/49.2	
Weight gain	−5.8/24.3	

## Data Availability

Data is available from the corresponding author upon reasonable request.

## Supplementary Materials

The following are available online at https://www.mdpi.com/article/10.3390/jcm10204653/s1, Table S1: Mean Dosage to organs at risk—complete data.
