# Supplementary material for: Reduction of Elective Radiotherapy Treatment Volume in Definitive Treatment of Locally Advanced Head and Neck Cancer—Comparison of a Prospective Trial with a Revised Simulated Contouring Approach
Source: J Clin Med. 2021 Oct 11;10(20):4653. doi: 10.3390/jcm10204653 (PMC8537676; doi:10.3390/jcm10204653)
Supplement: Supplementary file 1 [file jcm-10-04653-s001.zip › jcm-1388242-supplementary.pdf]

Table S1. Mean Dosage to organs at risk - complete data.

	Standart Treatment (ST)		Volume Reduced Treatment (VRT)		Significance *p*-Value
**Structure**	**Median**	**25/75% Quartile**	**Median**	**25/75% Quartile**	
PTV_56 Volume (cm^3)	1091.9	889.8/1268.2	750.3	607.2/884.9	$p < 0.001$
PTV_56 Dmean (Gy)	63.9	63.1/64.7	63.7	62.1/64.9	$p = 1.000$
PTV_64 Volume (cm^3)	754.3	625.1/1036.1	368.77	330.2/468.5	$p < 0.001$
PTV_64 Dmean (Gy)	66.9	66.0/67.7	69.3	68.5/69.6	$p = 1.000$
PTV_70 Volume (cm^3)	304.7	189.3/412.5	279.9	201.3/374.2	$p = 1.000$
PTV_70 Dmean (Gy)	70.7	70.5/70.9	70.7	70.2/70.9	$p = 1.000$
Ary carilage Dmean Dmean (Gy)	65.9	60.5/71.1	59.1	38.5/70.7	$p = 0.046$
BOT Dmean (Gy)	63.8	55.4/69.5	59.5	38.1/68.9	$p = 0.086$
Buccal mucosa Dmean (Gy)	32.5	25.1/40.9	30.2	22.3/35.1	$p = 0.037$
Carotid Artery Dmean (Gy)	62.4	60.5/64.7	56.2	50.6/59.5	$p < 0.001$
M. crycopharyngeus Dmean (Gy)	64.7	58.6/69.8	55.9	38.5/64.4	$p < 0.001$
EIM Dmean (Gy)	55.6	44.3/63.4	45.9	28.9/57.7	$p < 0.001$
Glomus caroticum Dmean (Gy)	67.7	65.2/69.9	65.3	62.9/68.1	$p < 0.001$
Supraglott Larynx Dmean (Gy)	68.9	65.2/70.9	66.6	59.3/70.1	$p = 0.046$
Glott Larynx Dmean (Gy)	64.7	50.9/69.6	55.5	33.9/69.5	$p < 0.001$
Larynx (supra +glott) Dmean (Gy)	68.6	62.5/70.6	61.1	51.6/70.2	$p < 0.001$
Superior PCM Dmean (Gy)	66.2	61.4/68.9	64.5	56.4/67.3	$p = 0.541$
Middle PCM Dmean (Gy)	70.1	67.4/70.9	68.1	65.4/70.3	$p = 0.020$
Inferior PCM Dmean (Gy)	68.3	63.8/71.1	62.6	48.9/70.5	$p < 0.001$
Temporomandubular joint Dmean (Gy)	20.1	16.6/21.8	20.6	18.7/22.7	$p = 1.000$
M. masseter Dmean (Gy)	29.7	26.2/34.1	27.8	24.9/32.2	$p = 0.710$
Plexus brachialis Dmean (Gy)	44.6	40.7/53.4	41.2	35.6/44.4	$p < 0.001$
Pterygoideus medialis Dmean (Gy)	59.3	50.6/64.9	53.2	47.9/59.7	$p = 0.099$
Pterygoideus lateralis Dmean (Gy)	38.3	33.4/42.9	35.7	31.6/37.9	$p = 1.000$
Softpalatine Dmean (Gy)	62.9	46.6/67.8	56.3	44.5/65.7	$p = 0.692$
M. temporalis Dmean (Gy)	10.1	7.6/13.5	11.5	9.5/12.5	$p = 1.000$
Zervical-Oesophagus	37.6	31.0/47.5	19.8	9.4/48.8	$p < 0.001$

Dmean (Gy)					
Lips Dmean (Gy)	22.7	18.2/29.3	20.1	13.8/26.7	$p < 0.001$
Mandibula Dmean (Gy)	42.0	35.7/52.0	33.5	27.9/44.2	$p < 0.001$
Oral cavity Dmean (Gy)	49.1	39.3/57.9	41.6	29.0/53.8	$p = 0.020$
Parotid Dmean (Gy)	32.4	28.5/39.2	32.5	28.1/36.9	$p = 1.000$
Sublingual Glandula Dmean (Gy)	40.1	26.0/64.9	31.6	15.9/52.5	$p = 0.143$
Submandibular Glandula Dmean (Gy)	66.6	60.3/69.5	56.8	50.6/67.6	$p < 0.001$
Thyroid Dmean (Gy)	58.9	56.8/61.4	45.7	33.2/53.5	$p < 0.001$

	Standart Treatment (ST)		Volume reduced treatment (VRT)		Significance p-value
Structure	Median	25%/75% quartile	Median	25%/75% quartile	
PTV_56 Volume (cm^3)	1091,9	889,8/1268,2	750,3	607,2/ 884,9	p<0.001
PTV_56 Dmean (Gy)	63,9	63,1/ 64,7	63,7	62,1/ 64,9	P=1.000
PTV_64 Volume (cm^3)	754,3	625,1/ 1036,1	368,77	330,2/ 468,5	p<0.001
PTV_64 Dmean (Gy)	66,9	66,0/ 67,7	69,3	68,5 / 69,6	P=1.000
PTV_70 Volume (cm^3)	304,7	189,3/ 412,5	279,9	201,3/ 374,2	P=1.000
PTV_70 Dmean (Gy)	70,7	70,5/ 70,9	70,7	70,2/ 70,9	P=1.000
Ary carilage Dmean Dmean (Gy)	65,9	60,5/ 71,1	59,1	38,5/ 70,7	P=0.046
BOT Dmean (Gy)	63,8	55,4/ 69,5	59,5	38,1/ 68,9	P=0.086

Buccal mucosa Dmean (Gy)	32,5	25,1/ 40,9	30,2	22,3/ 35,1	P=0.037
Carotid Artery Dmean (Gy)	62,4	60,5/ 64,7	56,2	50,6/ 59,5	p<0.001
M.crycopharyngeus Dmean (Gy)	64,7	58,6/ 69,8	55,9	38,5/ 64,4	p<0.001
EIM Dmean (Gy)	55,6	44,3/ 63,4	45,9	28,9/ 57,7	p<0.001
Glomus caroticum Dmean (Gy)	67,7	65,2/ 69,9	65,3	62,9/ 68,1	p<0.001
Supraglott Larynx Dmean (Gy)	68,9	65,2/ 70,9	66,6	59,3/ 70,1	P=0.046
Glott Larynx Dmean (Gy)	64,7	50,9/ 69,6	55,5	33,9/ 69,5	p<0.001
Larynx (supra +glott) Dmean (Gy)	68,6	62,5/ 70,6	61,1	51,6/ 70,2	p<0.001
Superior PCM Dmean (Gy)	66,2	61,4/ 68,9	64,5	56,4/ 67,3	P=0.541
Middle PCM Dmean (Gy)	70,1	67,4/ 70,9	68,1	65,4/ 70,3	P=0.020
Inferior PCM Dmean (Gy)	68,3	63,8/ 71,1	62,6	48,9/ 70,5	p<0.001
Temporomandubular joint Dmean (Gy)	20,1	16,6/ 21,8	20,6	18,7/ 22,7	P=1.000
M.masseter Dmean (Gy)	29,7	26,2/ 34,1	27,8	24,9/ 32,2	P=0.710
Plexus brachialis Dmean (Gy)	44,6	40,7/ 53,4	41,2	35,6/ 44,4	p<0.001

Pterygoideus medialis Dmean (Gy)	59,3	50,6/ 64,9	53,2	47,9/ 59,7	P=0.099
Pterygoideus lateralis Dmean (Gy)	38,3	33,4/ 42,9	35,7	31,6/ 37,9	P=1.000
Softpalatine Dmean (Gy)	62,9	46,6/ 67,8	56,3	44,5/ 65,7	P=0.692
M.temporalis Dmean (Gy)	10,1	7,6/ 13,5	11,5	9,5/ 12,5	P=1.000
Zervical-Oesophagus Dmean (Gy)	37,6	31,0/ 47,5	19,8	9,4/ 48,8	p<0.001
Lips Dmean (Gy)	22,7	18,2/ 29,3	20,1	13,8/ 26,7	p<0.001
Mandibula Dmean (Gy)	42,0	35,7/ 52,0	33,5	27,9/ 44,2	p<0.001
Oral cavity Dmean (Gy)	49,1	39,3/ 57,9	41,6	29,0/ 53,8	P=0.020
Parotid Dmean (Gy)	32,4	28,5/ 39,2	32,5	28,1/ 36,9	P=1.000
Sublingual Glandula Dmean (Gy)	40,1	26,0/ 64,9	31,6	15,9/ 52,5	P=0.143
Submandibular Glandula Dmean (Gy)	66,6	60,3/ 69,5	56,8	50,6/ 67,6	p<0.001
Thyroid Dmean (Gy)	58,9	56,8/ 61,4	45,7	33,2/ 53,5	p<0.001
